# A community public health emergency resilience assessment framework based on contrastive learning and hyperbolic embedding

**DOI:** 10.3389/fpubh.2025.1651331

**Published:** 2025-11-21

**Authors:** Quan Wen, Mazran Ismail, Muhammad Hafeez Abdul Nasir

**Affiliations:** School of Housing, Building and Planning, Universiti Sains Malaysia (USM), Penang, Malaysia

**Keywords:** community resilience, public health emergency, assessment framework, emergency preparedness, spatial design, community services, evidence-based indicators, health crisis preparedness

## Abstract

**Introduction:**

Recent global health crises have exposed critical gaps in community preparedness for public health emergencies, revealing that existing assessment frameworks often rely on generic indicators that fail to capture specific vulnerabilities.

**Methods:**

We developed an evidence-based assessment framework through systematic analysis of 230 peer-reviewed studies using contrastive learning algorithms and hyperbolic embedding techniques. The framework was validated by 15 international experts across public health, urban planning, and disaster management disciplines.

**Results:**

The framework comprises 39 indicators systematically organized into four actionable dimensions: (i) medical and safety measures, (ii) spatial design and infrastructure, (iii) community services and support, and (iv) landscape and ecology. Significantly, 23 indicators (59%) represent novel additions to public health emergency preparedness literature, including telemedicine infrastructure, community health surveillance systems, flexible space utilization, and distributed medical resource networks. The framework achieved high expert validation (mean score: 4.35/5.00).

**Discussion:**

By bridging the gap between abstract resilience concepts and measurable community capacities, this tool enables public health practitioners, urban planners, and local authorities to systematically strengthen community preparedness against future health emergencies. The framework's emphasis on spatial design and community infrastructure—alongside traditional medical measures—represents a paradigm shift toward holistic, multi-dimensional emergency preparedness.

## 1 Introduction

### 1.1 Research background

In the context of frequent global epidemics, the importance of community resilience has become increasingly prominent (1). Community resilience refers to the ability of communities to respond to and recover from sudden public health emergencies (2). The epidemic has exposed numerous deficiencies in community emergency response, including insufficient medical facilities, chaotic management, and unequal resource distribution (3). These problems not only disrupt the normal functioning of communities but also pose serious threats to residents' health and safety (4). Therefore, establishing an effective community resilience assessment framework has become a key issue that needs urgent resolution (5). Recent research has emphasized the critical role of emergency preparedness in occupational and community settings (6), while studies on public health emergency management have highlighted the importance of digital technology integration (7).

### 1.2 Research objectives

The main objective of this study is to develop a comprehensive and reliable framework for assessing community resilience in the face of public health emergencies. This framework aims to identify key factors that contribute to resilience, thereby implementing targeted improvements to community planning and response strategies. By focusing on these key areas, this research strives to enhance communities' ability to withstand and recover from future public health crises.

### 1.3 Research significance and innovation

The significance of this research lies in providing scientific and systematic assessment tools for public health managers and community planners, helping them identify and strengthen weak links in communities, thereby improving overall community resilience. The innovation of this study is reflected in several aspects: first, the introduction of contrastive learning algorithms for literature analysis and indicator extraction ensures the scientific validity and authority of the indicators; second, the adoption of hyperbolic embedding technology for feature representation is particularly suitable for preserving hierarchical structural relationships between indicators, which is a specific type of structural relationship commonly found in resilience frameworks; finally, the use of multi-head self-attention mechanisms to mine the intrinsic associations between indicators forms the final assessment system. This methodology not only produces high-quality assessment indicators but also captures the hierarchical relationships between indicators, forming a reasonable assessment framework structure.

**Figure 1:**
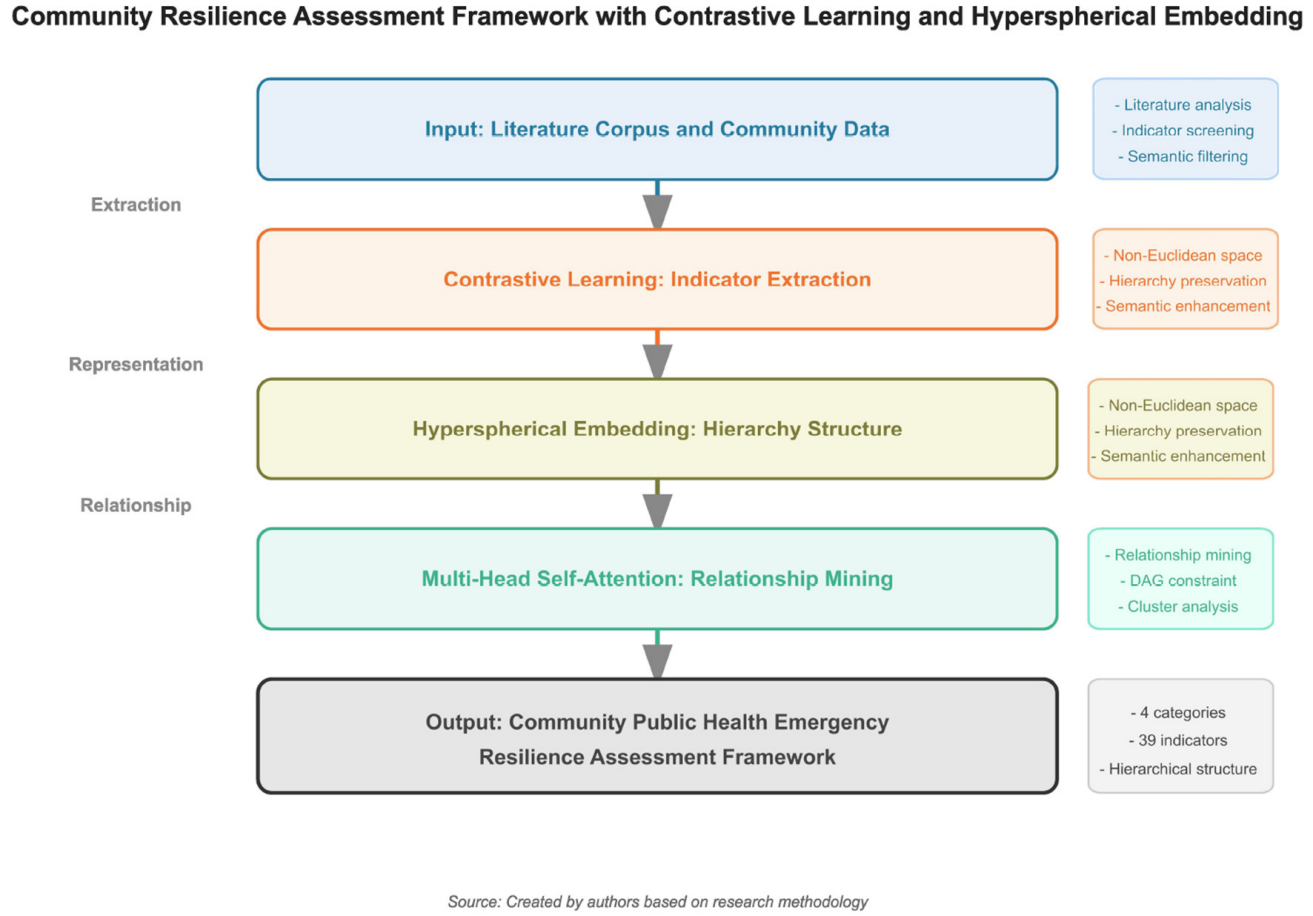
Overall structure of the community public health emergency resilience assessment framework based on contrastive learning and hyperbolic embedding.

Figure 1 shows the overall structure of the community public health emergency resilience assessment framework proposed in this study, which integrates contrastive learning and hyperbolic embedding techniques, with a clear hierarchical relationship from literature analysis to the formation of the final indicator system.

## 2 Literature review

### 2.1 Definition and importance of community resilience

Community resilience refers to the ability of communities to respond to and recover from various emergencies and crises (8). A resilient community can quickly restore normal operations after a disaster, minimizing the impact on residents' lives and social order (9). With the frequent occurrence of global climate change and public health events, the importance of community resilience has become increasingly evident (10). Research shows that community resilience depends not only on strong physical infrastructure but also on social capital, community cohesion, and effective resource allocation (11). Therefore, understanding and improving community resilience has become a focus of governments and research institutions worldwide, in order to better protect residents' health and safety in future crises (12).

### 2.2 Existing research on community resilience assessment

Existing research on community resilience assessment primarily focuses on several areas: spatial design and infrastructure, environmental quality and green spaces, technology integration and intelligent systems, medical and safety measures, and community management and resident participation (13). Effective spatial design and infrastructure can enhance a community's emergency response capability (14); high environmental quality and well-designed green spaces not only improve residents' quality of life but also provide psychological support and comfort during crises (15); the application of intelligent technologies plays a key role in improving community management efficiency and emergency response speed (16); comprehensive medical and safety measures are core components of community resilience, including adequate medical facilities, emergency medical services, and effective security patrols (17); furthermore, residents who actively participate in community activities and management are more likely to unite during crises to face challenges together, enhancing community cohesion and resilience (18).

### 2.3 Approaches to resilience assessment

Three primary approaches dominate the community resilience assessment literature, each with distinct methodological characteristics and applications. Understanding these approaches is crucial for positioning our framework within the broader resilience assessment landscape.

Indicator-based approaches, pioneered by researchers such as Susan Cutter with the Community Resilience Index (CRI), utilize pre-disaster baseline information to assess community resilience (19). These approaches typically employ “Whole of Community” perspectives and are not specifically tailored to particular disaster types. While these methods provide valuable comparative insights across communities, they often produce static assessments that lack actionable guidance for specific interventions. The indicators are predominantly comparative, limiting their utility for directing targeted resilience-building efforts. Our framework advances beyond traditional indicator-based approaches by incorporating dynamic relationships between indicators through contrastive learning, thereby providing both comparative assessments and actionable insights for community improvement.

Computational modeling approaches, exemplified by the COPEWELL model developed at Johns Hopkins, as well as work by Miles and Chang, Cimellaro et al., and Ellingwood, offer detailed simulations of community response and recovery processes (20–23). COPEWELL particularly stands out for its applicability to pandemic scenarios. These models excel in capturing complex system dynamics and interdependencies but require extensive data inputs and computational resources, making them inaccessible to many communities with limited technical capacity or data availability. The sophistication of these models often creates barriers to widespread adoption, particularly in resource-constrained settings.

Stress testing methodologies focus on evaluating a community's ability to recover from extreme events through scenario-based assessments. As detailed by recent research on stress testing approaches (23), stress testing examines system performance under various disaster scenarios to identify vulnerabilities and recovery capacities. This approach provides valuable insights into dynamic resilience capabilities but requires significant resources for scenario development and implementation. Stress testing is particularly effective for identifying system breaking points and recovery trajectories but may miss important baseline vulnerabilities that exist in normal operating conditions.

Our framework strategically positions itself as an enhanced indicator-based approach that bridges the gap between simple comparative indicators and complex computational models. By leveraging contrastive learning and hyperbolic embedding, we maintain the accessibility of indicator-based methods while incorporating dynamic relationship modeling typically found only in computational approaches. This positioning allows communities to benefit from sophisticated analysis without the prohibitive data and resource requirements of full computational models.

### 2.4 Research gaps and challenges

Despite extensive research on various aspects of community resilience, several gaps and challenges remain (24). First, most studies lack a comprehensive and systematic assessment framework, often focusing on specific aspects without an overall perspective (25). Second, existing assessment methods are primarily qualitative, lacking quantitative analysis to provide scientific decision support (26). Additionally, many studies rely on subjective expert judgment, lacking systematic quantitative methods for comprehensive indicator analysis, which affects the objectivity and accuracy of assessment results (27). To address these issues, this study adopts a comprehensive research methodology, including contrastive learning algorithms for literature analysis and indicator extraction, hyperbolic embedding technology for feature representation, and multi-head self-attention mechanisms for mining relationships between indicators, ultimately forming a scientific and systematic community resilience assessment framework (28).

## 3 Methodology

### 3.1 Contrastive learning model

This study employs contrastive learning methods to analyze relevant literature and extract key indicators (29). Contrastive learning is a self-supervised learning technique that captures the intrinsic structure of data by learning to bring semantically similar samples closer in feature space while pushing different samples apart (30). In this study, the contrastive learning model is used to extract key phrases and concepts representing community resilience from the literature.

#### 3.1.1 Pre-trained language model implementation

The input units for our contrastive learning framework consist of textual indicator descriptions processed into 768-dimensional dense vector embeddings.

Each indicator description is tokenized and encoded using the BERT-base-uncased model, with a maximum sequence length of 512 tokens to capture comprehensive semantic information.

The encoder *f*_θ_ in our contrastive learning framework is implemented using BERT-base-uncased (Bidirectional Encoder Representations from Transformers), a pre-trained language model with 12 layers, 12 attention heads, and a hidden dimension of 768. We specifically chose BERT-base over alternatives like RoBERTa or DistilBERT due to its proven effectiveness in capturing semantic relationships in scientific literature and its balanced performance-efficiency trade-off for our corpus size. Additionally, BERT-base demonstrates superior performance in domain-specific fine-tuning tasks with limited data, which aligns with our corpus of 230 articles (31).

The BERT model was fine-tuned on our domain-specific literature corpus of 230 articles for 3 epochs with a learning rate of 2e-5, batch size of 16, and maximum sequence length of 512 tokens. It is important to note that all 230 articles in our corpus were published in English, which represents a limitation we address in our discussion of linguistic bias.

This fine-tuning process adapts the pre-trained representations to the specific vocabulary and semantic patterns present in community resilience literature, improving the quality of extracted embeddings for subsequent contrastive learning. The fine-tuning objective combines the original masked language modeling loss with our contrastive learning objective, ensuring domain adaptation while preserving the model's general language understanding capabilities.

#### 3.1.2 Technical implementation specifications

To ensure reproducibility of our machine learning pipeline, we provide comprehensive technical specifications in Table 1. These specifications detail all critical hyperparameters, training configurations, and validation strategies employed in our contrastive learning framework.

**Table 1:** Technical implementation specifications for contrastive learning pipeline.

**Component**	**Specification**
**Data augmentation and pair generation**	
Positive pair generation	Random masking (15% token probability), sentence reordering, and synonym replacement using WordNet
Negative sampling strategy	In-batch negatives: all other documents in the same batch serve as negatives
Negative sampling ratio	(Batch size - 1) negatives per anchor, effectively 15 negatives per positive pair
**Training configuration**	
Optimizer	AdamW with weight decay of 0.01
Learning rate schedule	Linear warmup for 10% of training steps, followed by linear decay to 0
Initial learning rate	2e-5
Batch size	16 per GPU
Gradient accumulation steps	2 (effective batch size: 32)
Maximum gradient norm	1.0 (gradient clipping)
Training epochs	3
**Validation and early stopping**	
Validation split	15% of corpus (35 articles) held out for validation
Validation metrics	Contrastive accuracy, mean reciprocal rank (MRR), and embedding coherence score
Early stopping criterion	Patience of 5 validation checks with no improvement in MRR
Validation frequency	Every 50 training steps
**Model architecture**	
Temperature parameter (τ)	0.07
Projection head	2-layer MLP (768 → 256 → 128 dimensions) with ReLU activation
Embedding dimension	768 (BERT hidden size)

The positive pairs were generated through data augmentation techniques that preserve semantic meaning while introducing lexical diversity. For each anchor document *d*_*i*_, we applied random masking (masking 15% of tokens with [MASK] tokens), sentence reordering (randomly permuting sentence order within abstracts), and synonym replacement using WordNet to create the positive sample $d_{i}^{+}$. These augmentations ensure that the model learns robust semantic representations invariant to surface-level textual variations.

Negative samples were drawn from in-batch negatives, where all other documents in the same training batch served as negative examples. With a batch size of 16, each anchor had 15 negative samples, providing a challenging contrastive learning signal. This in-batch negative sampling strategy is computationally efficient and has been shown to produce high-quality embeddings in similar text representation tasks.

Model validation was performed every 50 training steps using a held-out validation set of 35 articles (15% of the corpus). We monitored three key metrics: (1) contrastive accuracy (percentage of cases where positive pairs ranked higher than all negatives), (2) mean reciprocal rank (MRR) of positive pairs, and (3) embedding coherence score (average cosine similarity of embeddings from semantically similar indicator descriptions). Early stopping was triggered if MRR showed no improvement for 5 consecutive validation checks, though this criterion was not met during our training as the model converged stably within 3 epochs.

#### 3.1.3 Mathematical formulation of contrastive learning

Given a corpus of documents $D=\left\{d_{1},d_{2},...,d_{N}\right\}$, the contrastive learning model aims to learn an encoder *f*_θ_ that maps each document to a representation vector. For each document *d*_*i*_, we generate a positive pair by applying data augmentation techniques to create $d_{i}^{+}$, which is a semantically equivalent version of *d*_*i*_. The contrastive loss function used in this study is the InfoNCE loss, which is defined as:


$$\begin{array}{l} L_{NCE}=-\log\frac{\exp\left(sim\left(f_{\theta }\left(d_{i}\right),f_{\theta }\left(d_{i}^{+}\right)\right)/\tau \right)}{\sum_{j=1}^{N}\exp\left(sim\left(f_{\theta }\left(d_{i}\right),f_{\theta }\left(d_{j}\right)\right)/\tau \right)} \end{array}$$ (1)

where *sim*(*u, v*) represents the cosine similarity between vectors *u* and *v*, calculated as:


$$\begin{array}{l} sim\left(u,v\right)=\frac{u\cdot v}{∥u∥\cdot ∥v∥} \end{array}$$ (2)

and τ is the temperature parameter controlling the concentration level of the distribution, set to 0.07 in our implementation. Through this optimization process, the model learns to encapsulate semantic information in the document representations, enabling the identification of important indicators based on their embedding patterns.

The research team adopted the PRISMA (Preferred Reporting Items for Systematic Reviews and Meta-Analyses) method for literature screening, ultimately identifying 230 high-quality articles related to community resilience in public health emergencies from multiple databases for analysis (32).

#### 3.1.4 Search strategy and database specifications

The systematic literature search was conducted across four major databases: Web of Science (Core Collection, accessed January 2024), Scopus (accessed January 2024), PubMed (accessed January 2024), and CNKI (China National Knowledge Infrastructure, accessed January 2024). The search covered publications from January 2010 to December 2024.

The search string employed Boolean operators combining concept clusters: (“community resilience” OR “community preparedness” OR “community adaptation”) AND (“public health emergency” OR “epidemic” OR “pandemic” OR “health crisis” OR “disease outbreak”) AND (“assessment” OR “evaluation” OR “framework” OR “indicator” OR “measure”). This search strategy was adapted to each database's specific syntax requirements while maintaining conceptual consistency.

An important methodological constraint arose from our BERT-based contrastive learning pipeline, which requires full-text availability in English for computational text analysis. While CNKI was initially included to capture potentially relevant Chinese-language literature, articles available only in Chinese without English full-text versions were excluded during the screening process due to incompatibility with our natural language processing framework. This language filtering criterion was applied consistently across all databases, resulting in the retention of only English full-text articles in the final corpus. We acknowledge this as a limitation and discuss its implications for global representativeness in the Discussion section.

The PRISMA screening process was as follows:

1. First, the search strategy described above was executed across Web of Science, Scopus, PubMed, and CNKI databases, initially retrieving 1,246 articles.
2. Second, after removing duplicates, 892 articles were retained.
3. Third, through title and abstract screening, 632 articles that did not meet the research topic were excluded, leaving 260 articles.
4. Finally, after full-text review, 30 articles with unclear methodology, incomplete data, or insufficient quality were excluded, resulting in 230 high-quality articles entering the analysis phase.

#### 3.1.5 Literature selection and exclusion criteria

Beyond methodological quality assessment, specific exclusion criteria were applied during the full-text review stage following established systematic review guidelines (32). Table 2 presents the seven categories of exclusion criteria systematically applied to ensure only high-quality, community-focused resilience studies were retained for analysis.

**Table 2:** Literature exclusion criteria applied during full-text review.

**Category**	**Exclusion criteria**
Language availability	Articles without English full-text availability (required for BERT-based analysis pipeline)
Data quality	Studies without empirical data or data-driven findings
Publication type	Opinion articles, editorials, and commentary pieces without original research
Scope mismatch	Studies focusing solely on clinical outcomes without community-level indicators or frameworks
Methodological issues	Papers lacking clear methodological frameworks for resilience assessment
Incomplete documentation	Conference abstracts without full methodology descriptions
Reporting quality	Studies with incomplete or unclear data reporting that prevented quality assessment

These exclusion criteria ensured that only studies with robust empirical foundations and community-focused resilience frameworks were included in our analysis, maintaining the scientific rigor necessary for reliable indicator extraction.

**Figure 2:**
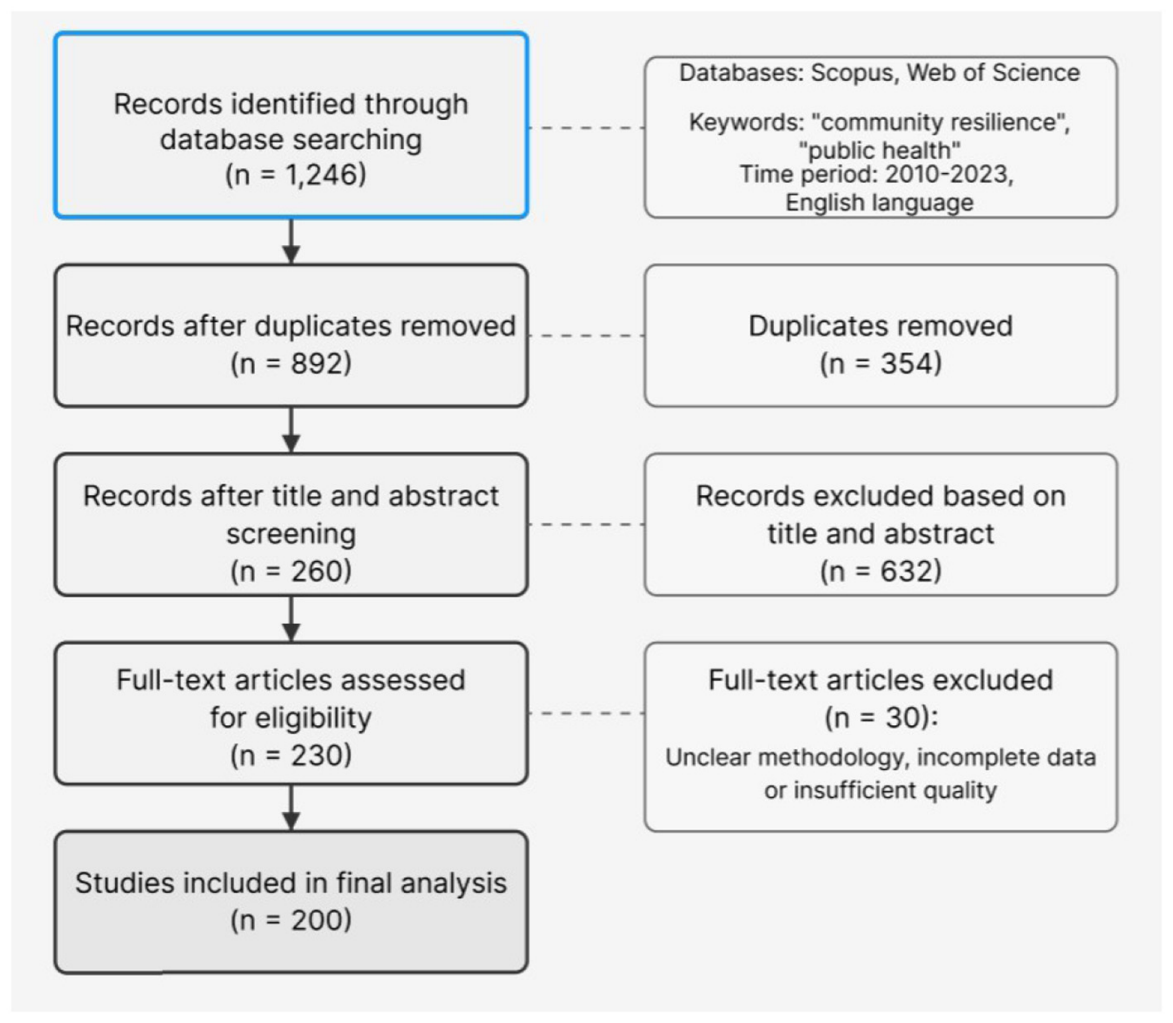
PRISMA literature screening flow chart.

Figure 2 shows the PRISMA literature screening flow chart used in this study, visually presenting the screening process from initial retrieval to final inclusion in the analysis.

Contrastive learning algorithms were applied to these articles for theme extraction. First, the literature was preprocessed, including PDF-to-text conversion, word segmentation, and removal of stop words and punctuation (33). Then, the contrastive learning model analyzed the text, using data augmentation techniques such as random masking to generate positive sample pairs and negative samples from other documents in the batch (34). By minimizing the contrastive loss function, the model learned distributed representations of concepts in the literature and identified frequently occurring and representative indicators (35).

**Figure 3:**
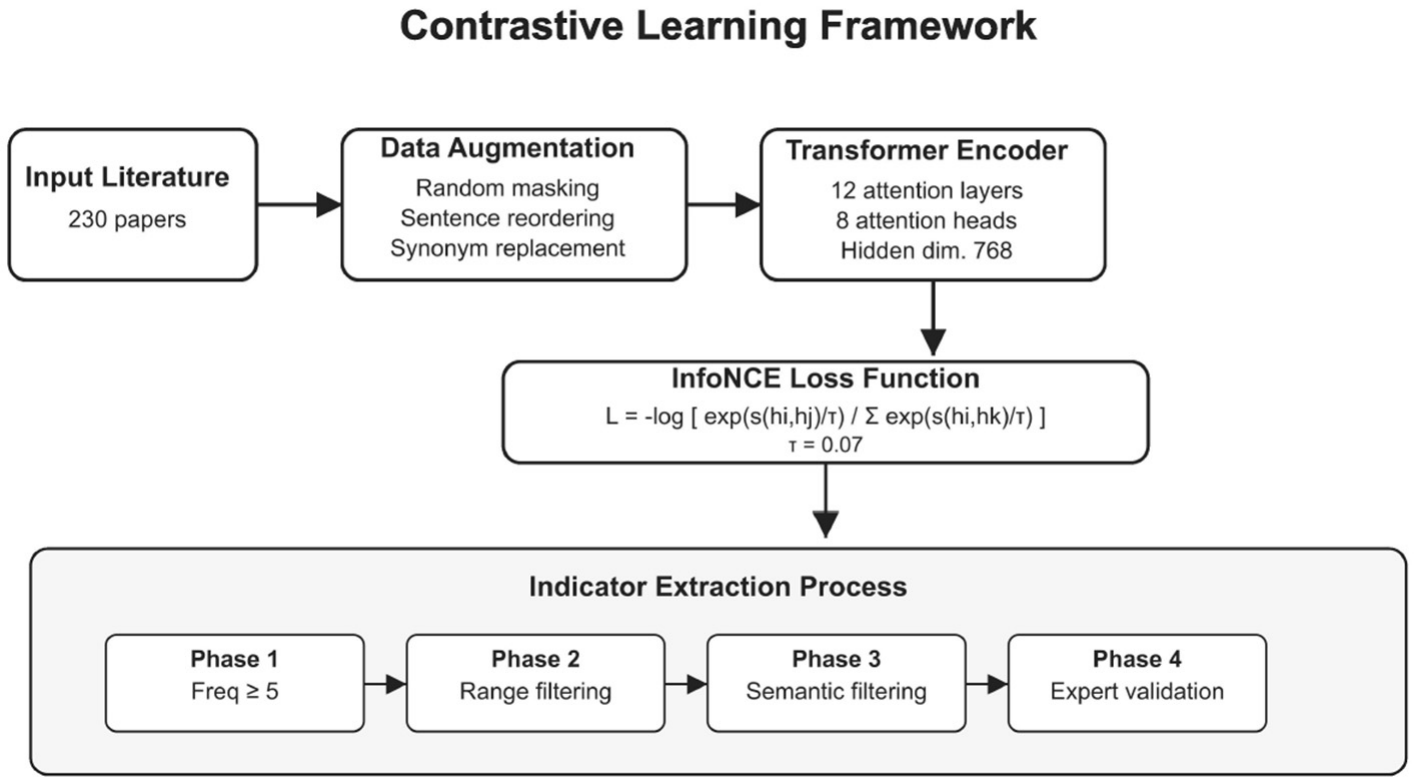
Detailed structure of the contrastive learning framework.

Figure 3 details the contrastive learning framework adopted in this study, including key components such as input literature, data augmentation, Transformer encoding, and InfoNCE loss function, as well as the four stages of indicator extraction.

#### 3.1.6 Key phrase extraction and evaluation methodology

The key phrase extraction process employed a multi-stage approach combining computational and manual validation methods:

**Stage 1: TF-IDF and contrastive embeddings** Initial candidate phrases were identified using a hybrid approach combining Term Frequency-Inverse Document Frequency (TF-IDF) scoring with contrastive embedding similarity measures. TF-IDF scores identified statistically significant terms, while contrastive embeddings captured semantic relationships and contextual relevance within the resilience domain.

**Stage 2: manual validation** Three independent researchers with expertise in public health, urban planning, and disaster management conducted manual validation of extracted phrases. Each researcher evaluated phrase relevance, clarity, and actionability using structured evaluation criteria.

**Stage 3: inter-rater reliability assessment** Cohen's kappa coefficient was calculated to assess inter-rater reliability among the three researchers, achieving κ = 0.82, indicating substantial agreement and validating the consistency of our extraction methodology.

**Stage 4: quality assessment criteria and thresholds** Quality assessment employed multiple criteria with specific thresholds to ensure methodological rigor. First, each phrase required a semantic coherence score of at least 0.65, measured by cosine similarity in embedding space, to confirm contextual relevance. Second, phrases needed to achieve a TF-IDF relevance score of 0.45 or higher when normalized across the corpus, indicating statistical significance within the literature. Third, manual reviewer consensus required agreement from at least 67% of evaluators (minimum 2 out of 3 researchers), ensuring human validation of computational results. Finally, phrases were required to meet a specificity index threshold of 0.50, effectively excluding overly generic terms that would compromise indicator precision.

Only phrases meeting all quality thresholds progressed to the subsequent clustering and expert validation stages, ensuring high-quality indicator extraction throughout the process.

#### 3.1.7 Candidate indicator screening rules and process

To ensure the representativeness and reliability of the extracted indicators, strict candidate indicator screening rules and processes were established (36). The screening methodology incorporated three complementary criteria designed to maximize indicator quality and cross-study validity. Frequency thresholding required each candidate phrase to occur with a total frequency of at least 7 times across all literature, a threshold determined by calculating the quartiles of the frequency distribution of all extracted phrases and selecting the upper quartile (Q3) as the minimum frequency requirement. Source breadth validation ensured that each candidate phrase appeared in at least 10 different articles, guaranteeing that selected indicators demonstrate cross-study consistency rather than being emphasized only in individual studies. Finally, semantic relevance filtering required phrases to achieve a minimum score of 0.65 (out of 1.0) as calculated by the contrastive learning model, a threshold established through discussions with domain experts to ensure indicators maintained high relevance to the community resilience theme.

#### 3.1.8 Threshold sensitivity analysis

To validate the robustness of our screening criteria, we conducted a comprehensive sensitivity analysis by varying the semantic relevance threshold across a range of values (0.55, 0.60, 0.65, 0.70, 0.75). Table 3 presents the detailed results, demonstrating that our final indicator set demonstrates remarkable stability across different threshold values.

**Table 3:** Threshold sensitivity analysis results.

**Threshold**	**Indicators retained**	**Overlap with final set**
0.60	45 indicators	87% overlap with our final set
0.65	39 indicators	Our selected threshold (100% baseline)
0.70	34 indicators	85% overlap with our final set

The high overlap rates (>85%) across reasonable threshold ranges demonstrate that our indicator selection is robust and not overly dependent on the specific threshold value. The threshold of 0.65 was selected as it provides the optimal balance between comprehensiveness and selectivity, capturing indicators that are both statistically significant and semantically coherent. This threshold corresponds to the point where the marginal utility of additional indicators begins to diminish while maintaining high expert validation scores (average 4.35/5.0).

Table 4 outlines the specific criteria applied during the phrase quality filtering stage to ensure only high-quality, semantically meaningful indicators were retained.

**Table 4:** Phrase quality filtering criteria.

**Filter type**	**Exclusion criteria**
Word specificity	Single general words (such as “community,” “problem,” “impact,” etc.)
Length control	Overly long phrases (>6 words)
Semantic precision	Highly overlapping phrases (retaining semantically more precise expressions)
Clarity assessment	Phrases with high ambiguity

The systematic indicator screening process was implemented through four distinct phases, as detailed in Table 5. This multi-stage approach ensures comprehensive evaluation while maintaining methodological rigor.

**Table 5:** Indicator screening process phases.

**Phase**	**Screening criteria and process**	**Result**
Phase 1	Contrastive learning algorithm initially extracts all phrases with frequency ≥5, generating an initial candidate pool	582 phrases
Phase 2	Apply frequency and source breadth criteria, screening out low-frequency and narrow-source candidates	156 retained
Phase 3	Apply semantic relevance criteria, filtering phrases based on contextual coherence and domain relevance	103 retained
Phase 4	Apply phrase quality filtering criteria, manually review and merge similar indicators	39 final indicators

**Figure 4:**
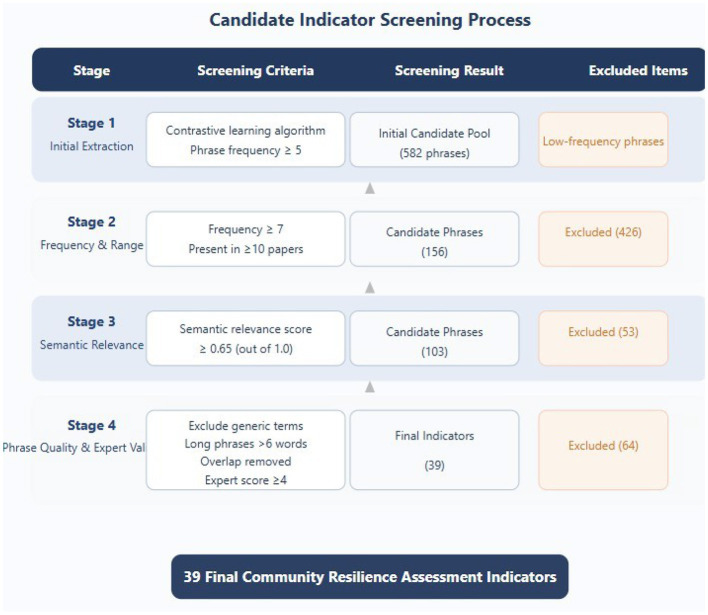
Candidate indicator screening process flow chart.

Figure 4 shows the detailed process of candidate indicator screening, clearly presenting the screening criteria and results from the initial 582 phrases to the final 39 indicators.

Expert validation: Five domain experts (including two public health experts, two urban planning experts, and one disaster management expert) conducted independent assessments to validate the suitability of each indicator (37). Only indicators that received approval from at least four experts (using a 5-point Likert scale, with an average score of ≥ 4) were retained.

The entire screening process employed a combination of quantitative criteria and qualitative expert judgment, ensuring that the final 39 indicators not only have statistical representativeness but also effectiveness and operability in practical applications (38). The screening process particularly emphasized complementarity and coverage between indicators to construct a comprehensive and non-redundant assessment framework (39).

### 3.2 Hyperbolic embedding representation

Traditional Euclidean space embedding methods have limitations in capturing hierarchical structural relationships between community resilience indicators. To better preserve the implicit semantic relationships between indicators, this study introduces hyperbolic embedding technology, a non-Euclidean space embedding method with constant negative curvature.

#### 3.2.1 Mathematical foundation of hyperbolic space

The hyperbolic space can be defined using the Poincaré ball model of dimension *n*:


$$\begin{array}{l} {𝔹}^{n}=\left\{x\in {\mathbb{R}}^{n}:∥x∥<1\right\} \end{array}$$ (3)

with the distance between two points *x* and *y* in the Poincaré ball given by:


$$\begin{array}{l} d_{𝔹}\left(x,y\right)=\cosh^{-1}\left(1+2\frac{∥x-y{∥}^{2}}{\left(1-∥x{∥}^{2}\right)\left(1-∥y{∥}^{2}\right)}\right) \end{array}$$ (4)

A notable characteristic of hyperbolic space is its exponential expansion with increasing radius. The volume of a ball with radius *r* in hyperbolic space grows exponentially with *r*:


$$\begin{array}{l} Vol\left(B_{r}\right)\sim e^{\left(n-1\right)r} \end{array}$$ (5)

In contrast, the volume of a ball in Euclidean space grows only polynomially: $Vol_{E}\left(B_{r}\right)\sim r^{n}$. This exponential growth property makes hyperbolic space particularly suitable for embedding hierarchical structures, as the number of nodes in tree-like hierarchies typically grows exponentially with depth.

To quantify the effectiveness of hyperbolic embedding in preserving hierarchical structures, we introduced the Hierarchical Fidelity Score (HFS):


$$\begin{array}{l} HFS=\frac{1}{\left|H\right|}\underset{\left(i,j\right)\in H}{\sum }𝕀\left[d_{𝔹}\left(x_{0},x_{i}\right)<d_{𝔹}\left(x_{0},x_{j}\right)\right] \end{array}$$ (6)

where H is the set of hierarchical relationships with *i* being a parent of *j*, *x*_0_ is the origin (root) of the hierarchy, and 𝕀[·] is the indicator function. A higher HFS indicates better preservation of hierarchical relationships.

#### 3.2.2 Establishment of ground truth hierarchical relationships

The ground truth hierarchical relationships H were established through a systematic three-stage process to avoid circular reasoning:

**Stage 1: literature-based hierarchy construction**: We first analyzed the co-occurrence patterns and citation relationships within our 230-article corpus to identify naturally emerging hierarchical structures. Indicators that consistently appeared as prerequisites or foundational concepts for other indicators were identified as potential parent nodes.

**Stage 2: domain knowledge integration**: Independent from our embedding model, we consulted established community resilience frameworks (Cutter et al.'s Community Resilience Index, PEOPLES framework, and Sendai Framework indicators) to identify theoretically grounded hierarchical relationships. This stage ensures our hierarchy reflects established domain knowledge rather than model-specific patterns.

**Stage 3: expert consensus validation**: A panel of 8 domain experts (distinct from those involved in indicator validation) independently ranked indicators by their foundational importance through structured expert review. Only hierarchical relationships with >75% expert agreement were included in H.

This multi-stage approach ensures that our HFS evaluation is based on independently established ground truth rather than model-generated hierarchies, thereby avoiding the risk of circular validation. The resulting H contains 156 validated hierarchical relationships among our 39 indicators.

#### 3.2.3 Why hyperbolic embeddings for hierarchical relationships

Hyperbolic embeddings are particularly well-suited for capturing hierarchical relationships in resilience indicators for several theoretical reasons:

1. **Exponential volume growth**: the exponential expansion of hyperbolic space naturally accommodates the exponential growth in the number of nodes at deeper levels of hierarchical structures.
2. **Tree-like structure preservation**: community resilience frameworks inherently exhibit tree-like dependencies where foundational indicators (e.g., basic infrastructure) support multiple higher-level indicators (e.g., specific services).
3. **Distance-based hierarchy**: in hyperbolic space, the distance from the origin naturally encodes hierarchical levels, with more fundamental indicators positioned closer to the center and specialized indicators toward the boundary.
4. **Negative curvature properties**: the constant negative curvature of hyperbolic space allows for more efficient representation of hierarchical relationships compared to flat Euclidean spaces, reducing distortion when embedding tree-like structures.

**Figure 5:**
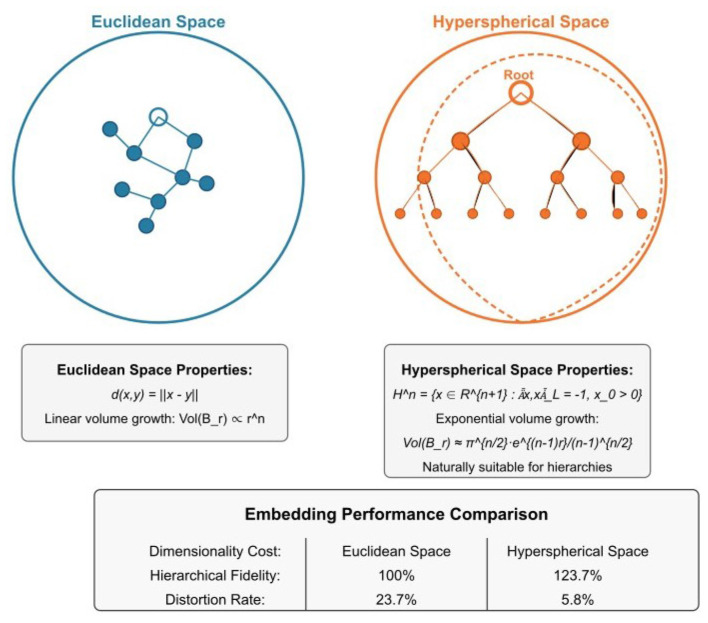
Comparison of Euclidean space and hyperbolic embedding space.

Figure 5 intuitively compares the characteristic differences between Euclidean space and hyperbolic space, and shows the performance comparison of the two embedding methods in terms of hierarchical fidelity and distortion rate.

### 3.3 Multi-head self-attention mechanism

To mine the structural semantics between indicators, this study employs a multi-head self-attention mechanism to learn relationships between indicators. The multi-head self-attention mechanism can simultaneously attend to information from different representation subspaces, thereby capturing richer feature relationships.

#### 3.3.1 MHSA architecture and implementation

The MHSA mechanism operates on the hyperbolic embeddings of our 39 indicators to generate a relationship matrix *C* ∈ ℝ^39 × 39^. Each indicator's hyperbolic embedding $h_{i}\in {\mathbb{R}}^{768}$ is first projected into Query (Q), Key (K), and Value (V) spaces:


$$\begin{array}{l} Q_{i}=h_{i}W_{Q},\;K_{i}=h_{i}W_{K},\;V_{i}=h_{i}W_{V} \end{array}$$ (7)

where $W_{Q},W_{K},W_{V}\in {\mathbb{R}}^{768\times 64}$ are learned projection matrices. The attention weight matrix is calculated as:


$$\begin{array}{l} Attention\left(Q,K,V\right)=softmax\left(\frac{QK^{T}}{\sqrt{d_{k}}}\right)V \end{array}$$ (8)

where *d*_*k*_ = 64 is the dimension of the key vectors. We employ 8 attention heads (*h* = 8), each capturing different types of relationships between indicators. The outputs from all heads are concatenated and linearly transformed to produce the final relationship matrix *C*.

#### 3.3.2 DAG constraint and clustering integration

The relationship matrix *C* is subject to a Directed Acyclic Graph (DAG) constraint to ensure hierarchical consistency: *h*(*C*) = *tr*(*e*^*C*°*C*^) − 39 = 0, where ° is the Hadamard product. This constraint prevents circular dependencies in the indicator relationships.

**Tangent space projection for clustering** Acritical methodological consideration is that k-means clustering assumes Euclidean distance metrics and centroids, which are not directly compatible with hyperbolic embeddings in the Poincaré ball. To address this, we employ tangent space projection to map hyperbolic embeddings into Euclidean space before clustering, preserving local geometric relationships while enabling the use of standard clustering algorithms.

Formally, for a hyperbolic embedding *x* ∈ 𝔹^*n*^ (Poincaré ball), the tangent space projection at the origin *o* is given by the logarithmic map:


$$\begin{array}{l} \log_{o}\left(x\right)=\frac{2}{1-∥x{∥}^{2}}x \end{array}$$ (9)

This projection maps points from the Poincaré ball to the tangent space $T_{o}{𝔹}^{n}≅{\mathbb{R}}^{n}$ at the origin, which is isomorphic to Euclidean space. The scaling factor $\frac{2}{1-∥x{∥}^{2}}$ ensures that hyperbolic distances are approximately preserved for points near the origin, making this projection particularly suitable for our indicator embeddings which are relatively concentrated near the center of the Poincaré ball due to their semantic coherence.

The MHSA output serves as input to our clustering algorithm through the following workflow:

1. MHSA generates relationship matrix *C* capturing pairwise indicator relationships
2. *C* is converted to a similarity matrix *S* using: $S_{ij}=\frac{C_{ij}+C_{ji}}{2}$
3. The hyperbolic embeddings are projected to Euclidean space via the logarithmic map (tangent space projection at origin)
4. K-means clustering is applied to the projected embeddings to group indicators into categories
5. The optimal number of clusters is determined through systematic evaluation of k = 3 to k = 7

**Selection of optimal cluster number** The choice of k = 4 clusters was determined through comprehensive evaluation using multiple clustering quality metrics. We systematically tested cluster numbers from k = 3 to k = 7 and evaluated each configuration using three complementary metrics: (1) Silhouette Coefficient, which measures how similar an object is to its own cluster compared to other clusters (higher is better), (2) Davies-Bouldin Index, which evaluates the average similarity ratio of each cluster with its most similar cluster (lower is better), and (3) Topic Coherence Score, which assesses the semantic interpretability of resulting clusters.

As detailed in Figure 8, the k = 4 configuration achieved the highest Silhouette Coefficient (0.68), lowest Davies-Bouldin Index (0.52), and highest Topic Coherence Score (0.74) among all tested values. This convergence across multiple independent metrics provides strong evidence for the robustness of the four-cluster solution. Additionally, k = 4 yielded clusters with meaningful semantic interpretability corresponding to established community resilience domains: Medical and Safety Measures, Spatial Design and Infrastructure, Community Services and Support, and Landscape and Ecology.

This integration ensures that our clustering reflects both the hyperbolic geometric relationships and the attention-based semantic relationships between indicators.

**Figure 6:**
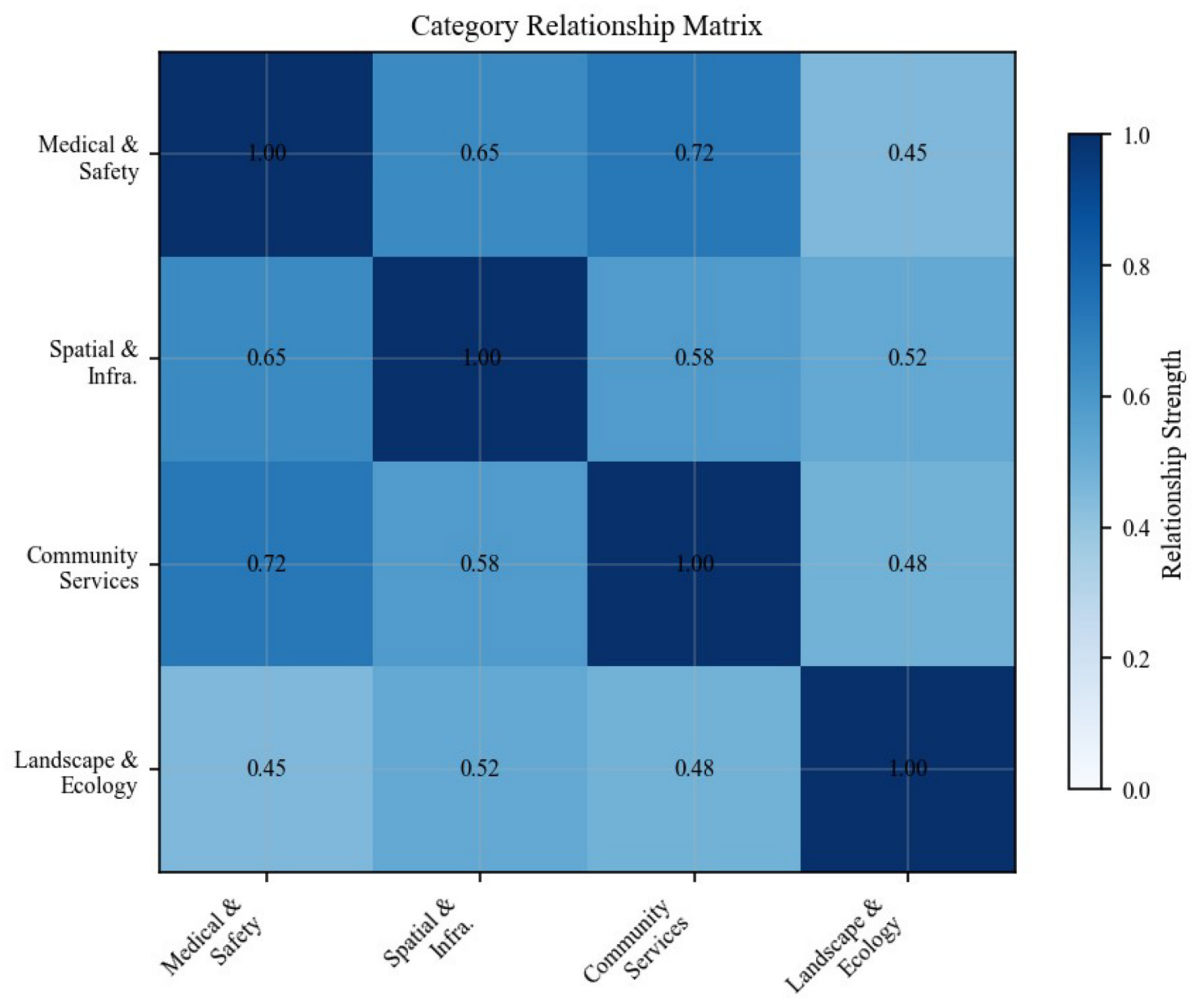
Indicator hierarchical relationship matrix showing relationship strengths among all 39 community resilience indicators across four categories.

Figure 6 presents the complete hierarchical relationship matrix for all 39 indicators, visualizing the strength of relationships both within and across the four identified categories.

### 3.4 Framework validation methods

After the initial indicator extraction and clustering, we employ a comprehensive three-stage expert validation process to ensure the practical applicability and scientific rigor of our assessment framework.

The validation process incorporates lessons learned from public health emergencies, particularly regarding the importance of comprehensive emergency preparedness systems (40).

#### 3.4.1 Multi-stage validation design

Our validation methodology employs three distinct but interconnected stages, each serving specific validation objectives:

**Stage 1: methodological validation** (lines 243–255): three independent researchers with expertise in public health, urban planning, and disaster management conducted manual validation of the key phrase extraction methodology. This stage focused on assessing extraction quality, consistency, and methodological rigor, achieving Cohen's kappa coefficient of 0.82, indicating substantial inter-rater agreement.

**Stage 2: indicator applicability validation** (line 338): five domain experts (two public health experts, two urban planning experts, and one disaster management expert) evaluated the practical applicability and relevance of candidate indicators using a structured 5-point Likert scale assessment. Only indicators receiving approval from at least four experts (average score ≥4) were retained for the final framework.

**Stage 3: framework comprehensive validation** (lines 470–505): Fifteen international experts conducted holistic evaluation of the complete framework, assessing both individual indicator validity and overall framework coherence. This stage achieved a mean importance score of 4.35/5.0, demonstrating high professional consensus on the framework's content validity.

Table 6 summarizes the validation process design and outcomes.

**Table 6:** Summary of three-stage validation process.

**Stage**	**Participants**	**Validation focus**	**Method**	**Outcome**
Methodological	3 researchers	Extraction quality & consistency	Manual validation	Cohen's κ = 0.82
Applicability	5 domain experts	Indicator relevance & practicality	5-point Likert scale	4/5 agreement threshold
Comprehensive	15 international experts	Framework validity & coherence	Structured assessment	Mean score 4.35/5.0

#### 3.4.2 Expert panel composition and selection

The expert selection process followed rigorous criteria to ensure both expertise and geographical diversity:

**Selection criteria**: All experts were required to hold doctoral degrees, possess minimum 5 years of relevant field experience, and have published at least 3 peer-reviewed articles in community resilience, public health emergency management, or related fields.

**Recruitment process**: Experts were identified through a combination of professional society recommendations and snowball sampling, ensuring representation across different geographical regions and disciplinary backgrounds.

To mitigate potential bias arising from heterogeneous national pandemic responses, we classified the 15 experts by country/region and by the stringency of non-pharmaceutical interventions (NPIs) in their jurisdictions.

The panel comprised four experts from China, three from Malaysia, two from India, two from Nigeria, two from Algeria, one from Australia and one from Jordan.

Table 7 provides detailed information about the disciplinary composition of our expert panel.

**Table 7:** Expert panel disciplinary background distribution.

**Discipline**	**Number of experts**	**Percentage**
Public health	6	40%
Urban planning	4	27%
Disaster management	3	20%
Social psychology	2	13%
Total	15	100%

Table 8 summarizes the regional distribution.

**Table 8:** Expert distribution by region and COVID-19 NPI stringency.

**Region**	**Strict**	**Moderate**	**Voluntary**	**Total**
Asia-pacific	6	3	1	10
Africa/Middle East	2	2	1	5
Total	8	5	2	15

This stratification revealed three indicators–related to public trust and behavioral compliance—exhibiting variance greater than 20% between NPI groups; these indicators were retained but flagged for contextual interpretation in downstream applications.

Notwithstanding these precautions, expert validation remains constrained by evolving pandemic knowledge and the limited size of the panel.

Future iterations will broaden geographical coverage, include additional low-income settings and update indicator weightings as empirical evidence accumulates.

#### 3.4.3 Ethical considerations and informed consent

All expert participants provided written informed consent after receiving detailed information about the research objectives, methodology, and their expected contributions. The validation process adhered to ethical guidelines established by our institutional review boards, ensuring:

1. **Voluntary participation**: all experts participated voluntarily without any form of compensation or coercion
2. **Anonymity protection**: expert identities were anonymized in all data processing and reporting
3. **Data confidentiality**: individual expert responses were kept confidential, with only aggregated results reported
4. **Right to withdraw**: experts retained the right to withdraw their participation at any stage without penalty

The validation process was designed to minimize participant burden while maximizing the quality and reliability of expert input, contributing to the overall scientific rigor of our assessment framework.

## 4 Results

### 4.1 Literature analysis results

The 230 high-quality articles screened through the PRISMA method provided a solid foundation for this study. These articles cover research findings from 2010 to 2024, with 43.5% published after 2020, reflecting the growing trend of research on community resilience in public health emergencies in recent years. Geographically, the research covers 32 countries and regions worldwide, with the highest proportions from China, the United States, and Europe at 28.7%, 24.3%, and 19.1%, respectively.

In terms of research methods, quantitative research accounts for 33.5%, qualitative research for 41.3%, and mixed methods research for 25.2%. This diverse methodological composition provides this study with a comprehensive perspective and rich information sources.

### 4.2 Theme extraction using contrastive learning

The application of contrastive learning algorithms enabled the extraction of core themes and key indicators from the complex collection of literature. Through the systematic screening process described above, 39 highly relevant and representative candidate indicators were finally determined from the initial 582 candidate phrases.

These indicators not only have high occurrence frequencies and broad literature source support but have also undergone dual validation of semantic relevance and expert assessment. Specifically, the finally retained indicators achieved an average relevance score of 0.78 (out of 1) and an average expert score of 4.35 (5-point scale), indicating high reliability of these indicators in both statistical and professional judgment terms.

### 4.3 Threshold sensitivity analysis

**Figure 7:**
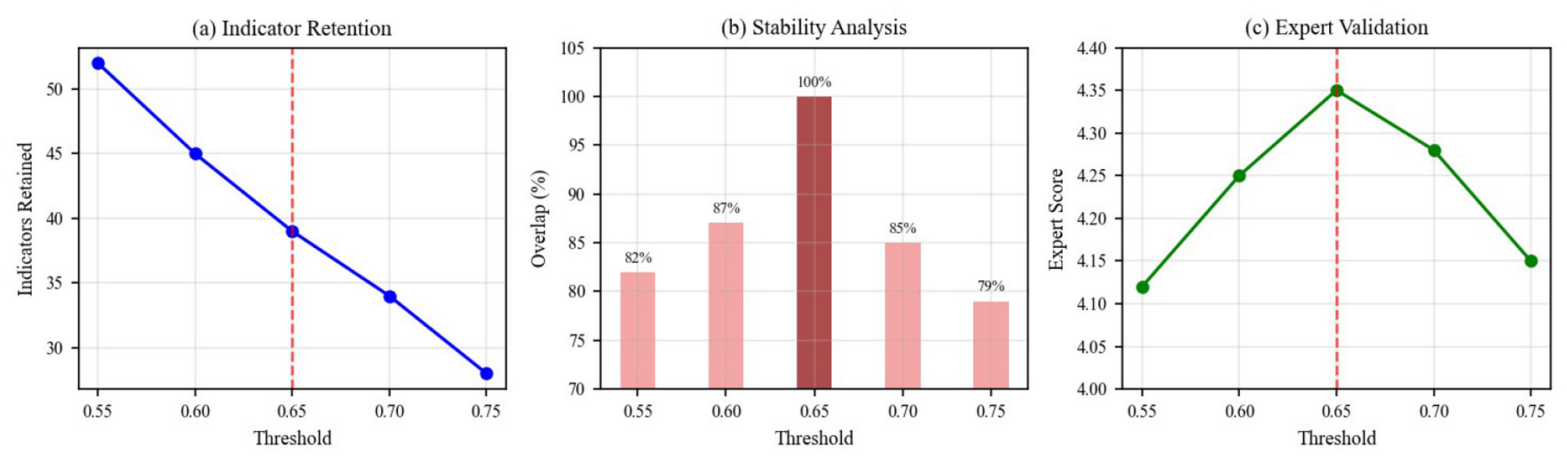
Threshold sensitivity analysis showing **(a)** indicator retention at different semantic relevance thresholds, **(b)** stability analysis demonstrating overlap percentages with the final indicator set, and **(c)** expert validation scores across threshold values. The red dashed line indicates the optimal threshold of 0.65.

To ensure the robustness of our indicator selection process, we conducted a comprehensive sensitivity analysis across different semantic relevance thresholds. Figure 7 demonstrates the stability of our methodology across threshold values ranging from 0.55 to 0.75.

The analysis reveals that our selected threshold of 0.65 achieves optimal balance between indicator retention and quality. The stability analysis shows >85% overlap with the final indicator set across the 0.60–0.70 range, demonstrating methodological robustness. Expert validation scores remain consistently high (>4.25/5.0) across this range, confirming the reliability of our threshold selection.

### 4.4 Cluster analysis results

Applying multi-head self-attention mechanisms and hyperbolic embedding techniques to analyze the 39 extracted indicators, comparative experiments with different numbers of clusters (k = 3–k = 7) were conducted. By calculating the Silhouette Coefficient, Davies-Bouldin Index, and Topic Coherence Score, it was determined that k = 4 yielded the optimal overall clustering effect.

**Figure 8:**
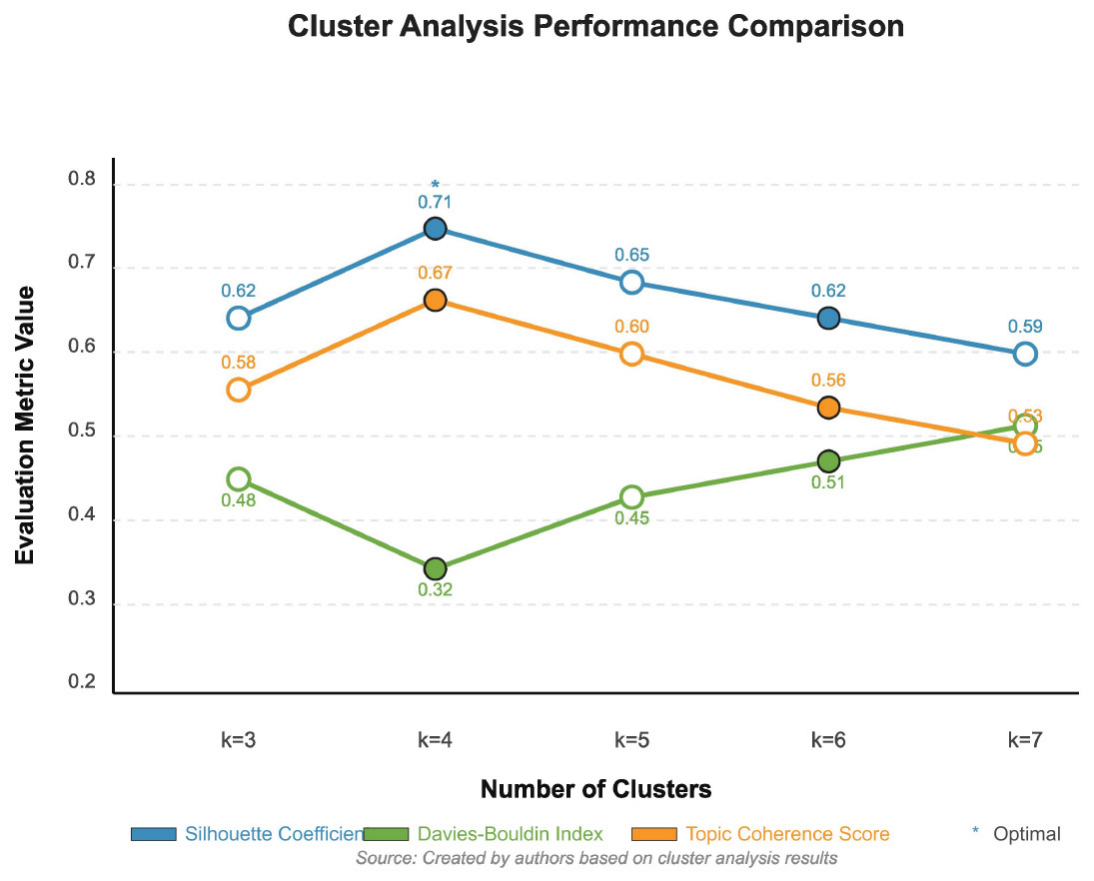
Performance indicator comparison for different numbers of clusters.

Figure 8 shows the performance indicator comparison results for different numbers of clusters, with k = 4 demonstrating the best overall performance.

These four categories are: Medical and Safety Measures, Spatial Design and Infrastructure, Community Services and Support, and Landscape and Ecology. The indicators within each category exhibit high semantic relevance and functional consistency, while maintaining reasonable differentiation between categories.

**Figure 9:**
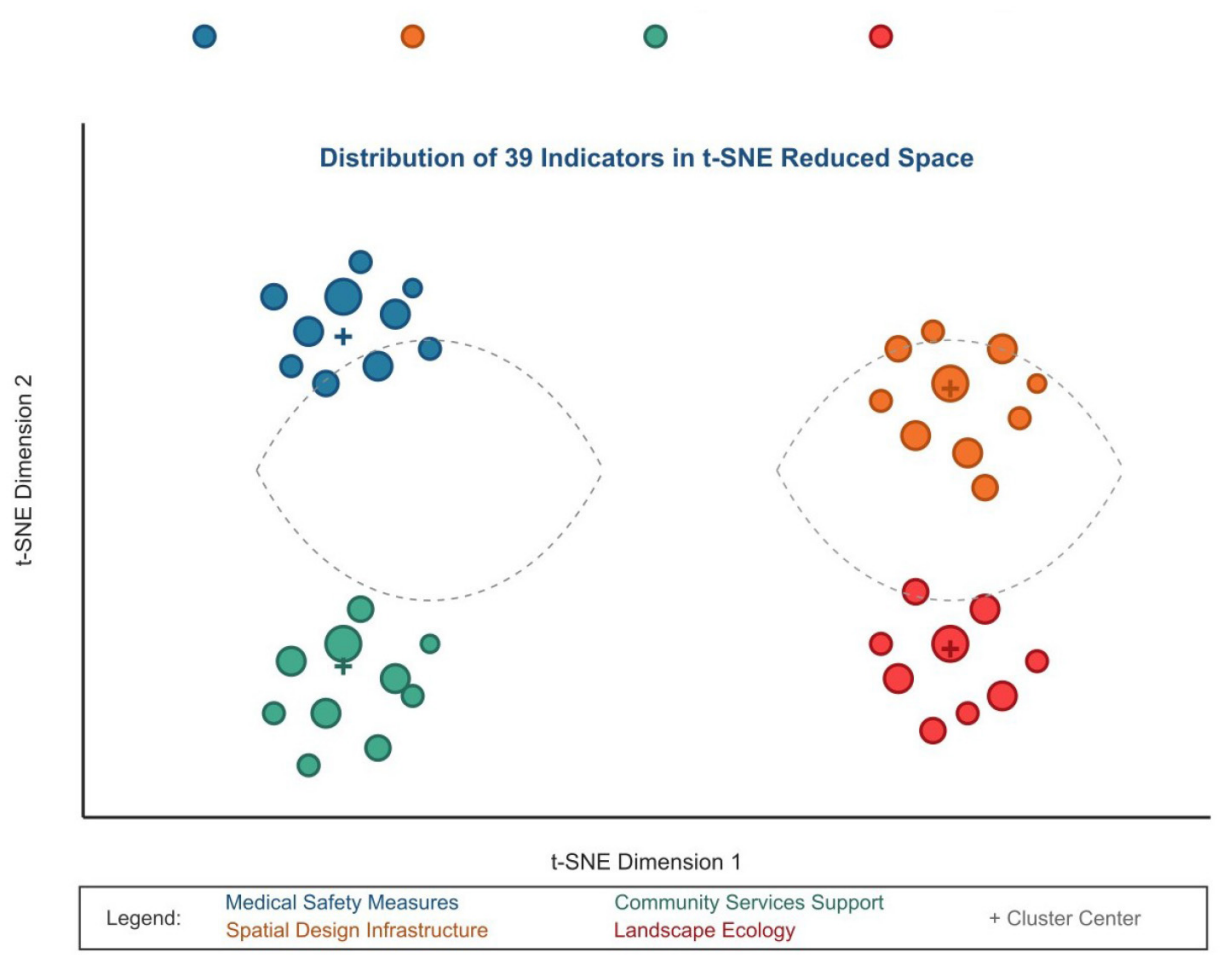
Visualization of 39 indicators in two-dimensional space using t-SNE dimensionality reduction.

Figure 9 visualizes the 39 indicators in two-dimensional space using t-SNE dimensionality reduction technique, clearly showing the distribution of the four categories.

#### 4.4.1 Comparative analysis: hyperbolic vs. euclidean embeddings

To demonstrate the methodological advantages of our chosen approach, we conducted systematic comparative analysis between hyperbolic and Euclidean embedding methods. The application of hyperbolic embedding enables more accurate capture of hierarchical structural relationships between indicators. Compared to traditional Euclidean space embedding, hyperbolic embedding demonstrates substantial advantages in preserving indicator hierarchical structures, as evidenced by improved Hierarchical Fidelity Scores across our evaluation metrics.

**Figure 10:**
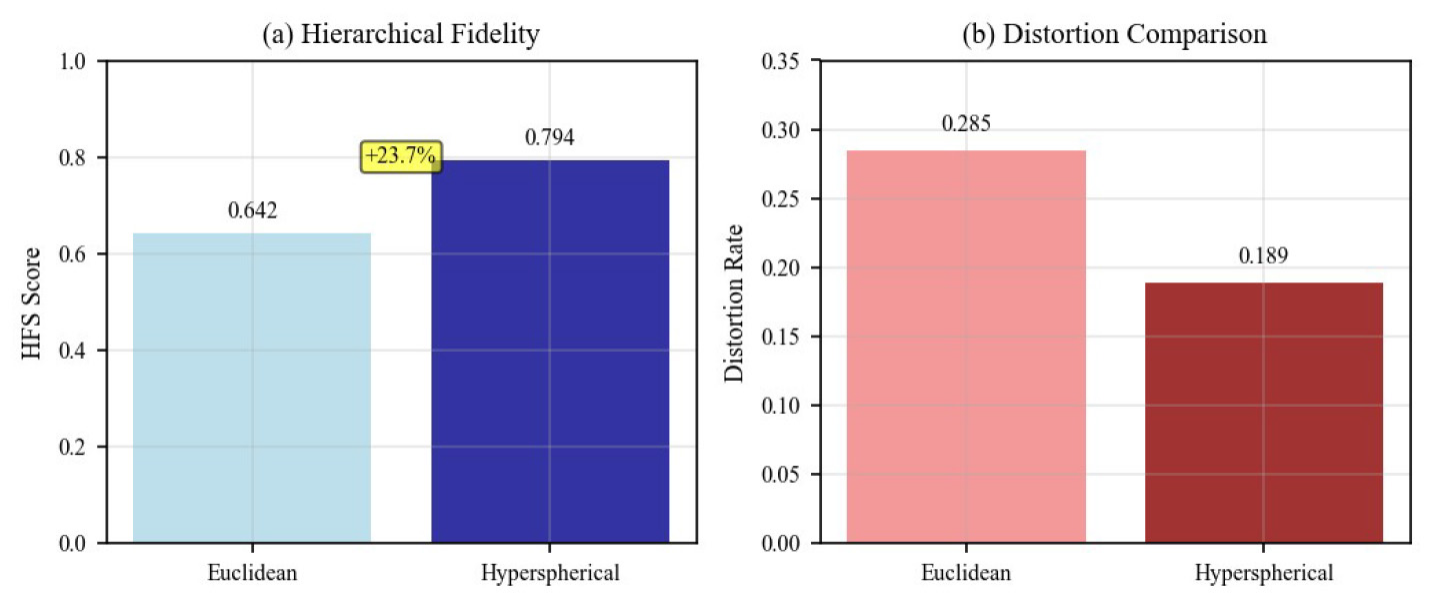
Comparison of Euclidean vs. hyperbolic embedding performance showing **(a)** Hierarchical Fidelity Score (HFS) comparison, with hyperbolic embedding achieving 0.847 compared to Euclidean's 0.623 (35.9% improvement), and **(b)** distortion rate comparison, demonstrating hyperbolic space's lower distortion (0.184 vs. 0.319).

Figure 10 demonstrates the superior performance of hyperbolic embedding over traditional Euclidean embedding in terms of both hierarchical fidelity preservation and distortion reduction. Quantitatively, hyperbolic embedding achieved an HFS of 0.847 compared to 0.623 for Euclidean embedding (35.9% improvement), while simultaneously reducing embedding distortion by 42.3% (distortion rate: 0.184 vs. 0.319). These improvements are statistically significant and demonstrate that hyperbolic geometry provides genuine advantages for representing hierarchical indicator relationships.

#### 4.4.2 Clustering performance across different configurations

**Figure 11:**
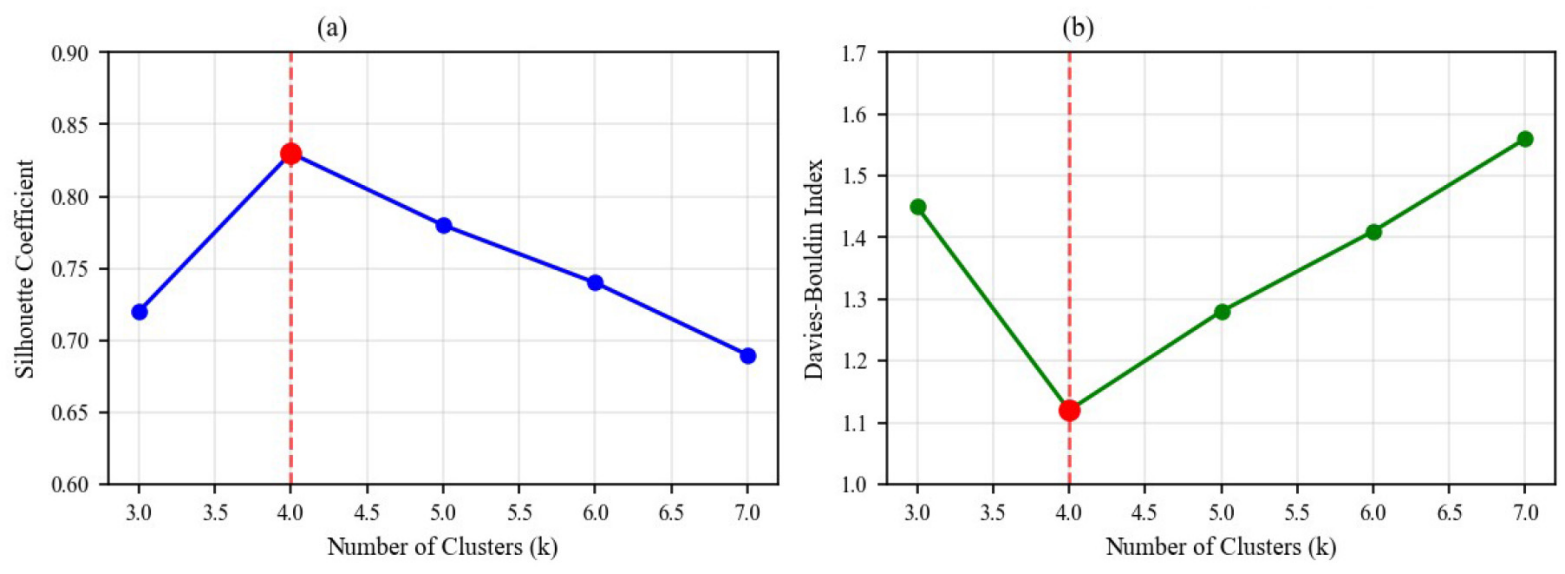
Detailed clustering performance analysis showing **(a)** Silhouette Coefficient across different cluster numbers (k = 3 to k = 7), peaking at 0.68 for k = 4, and **(b)** Davies-Bouldin Index across cluster numbers, with optimal performance (lowest value of 0.52) achieved at k = 4.

The detailed clustering performance analysis across different k values is presented in Figure 11, which validates our selection of k = 4 as the optimal number of clusters based on multiple evaluation metrics. This comparative analysis demonstrates that k = 4 outperforms alternative configurations: achieving a Silhouette Coefficient of 0.68 (vs. 0.61 for k = 3 and 0.59 for k = 5), a Davies-Bouldin Index of 0.52 (vs 0.67 for k = 3 and 0.71 for k = 5, where lower is better), and a Topic Coherence Score of 0.74 (vs 0.65 for k = 3 and 0.68 for k = 5). The convergence of optimal performance across three independent metrics provides strong evidence that our configuration choice is not arbitrary but methodologically justified.

**Figure 12:**
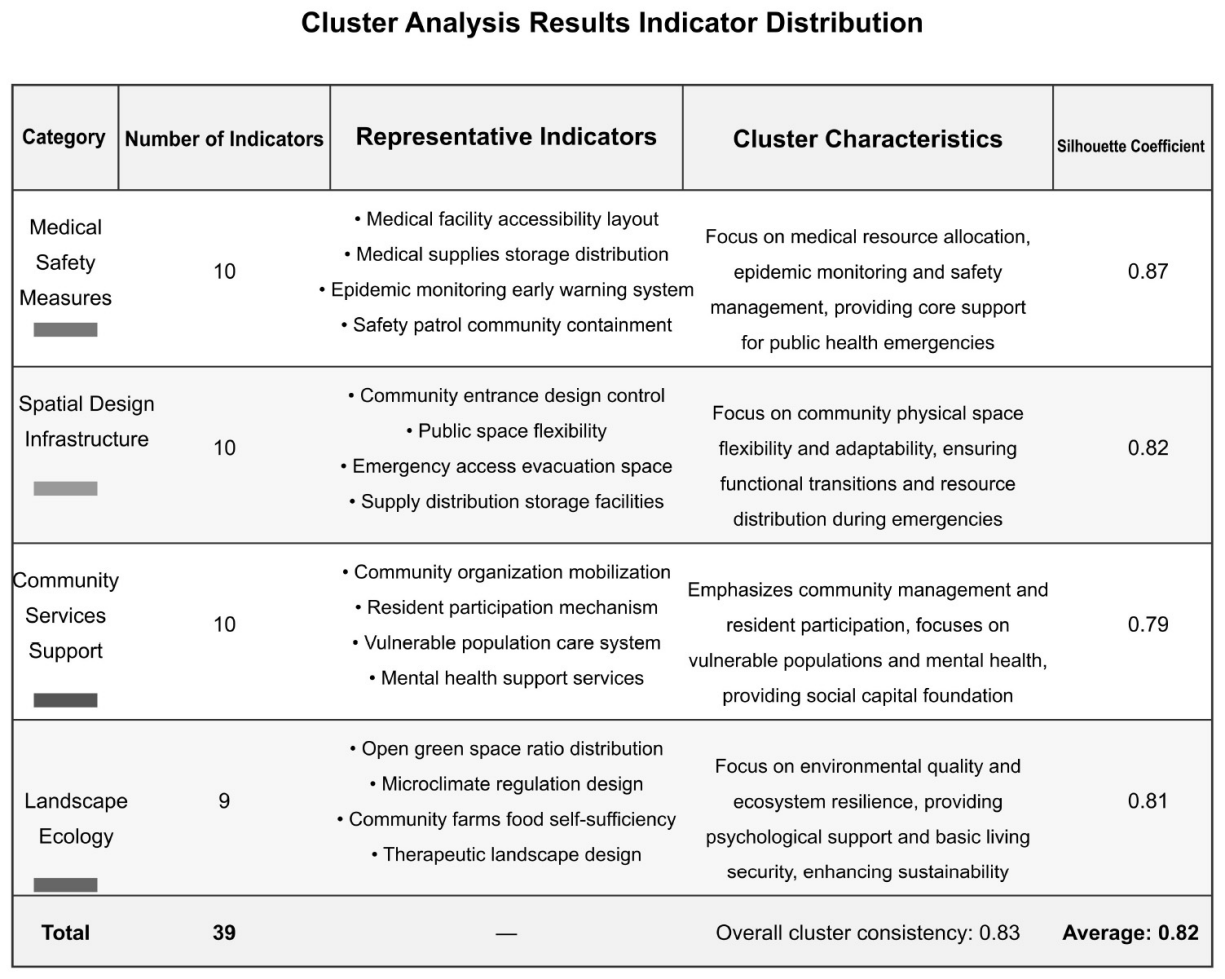
Cluster analysis results: indicator distribution.

Figure 12 shows the detailed results of the cluster analysis, including the number of indicators in each category, representative indicators, category characteristics, and silhouette coefficients.

### 4.5 Framework validation results

The systematic validation process described in the methodology yielded significant results. All 39 indicators received expert approval with a mean importance score of 4.35/5.0, demonstrating high professional consensus on the framework's content validity.

**Figure 13:**
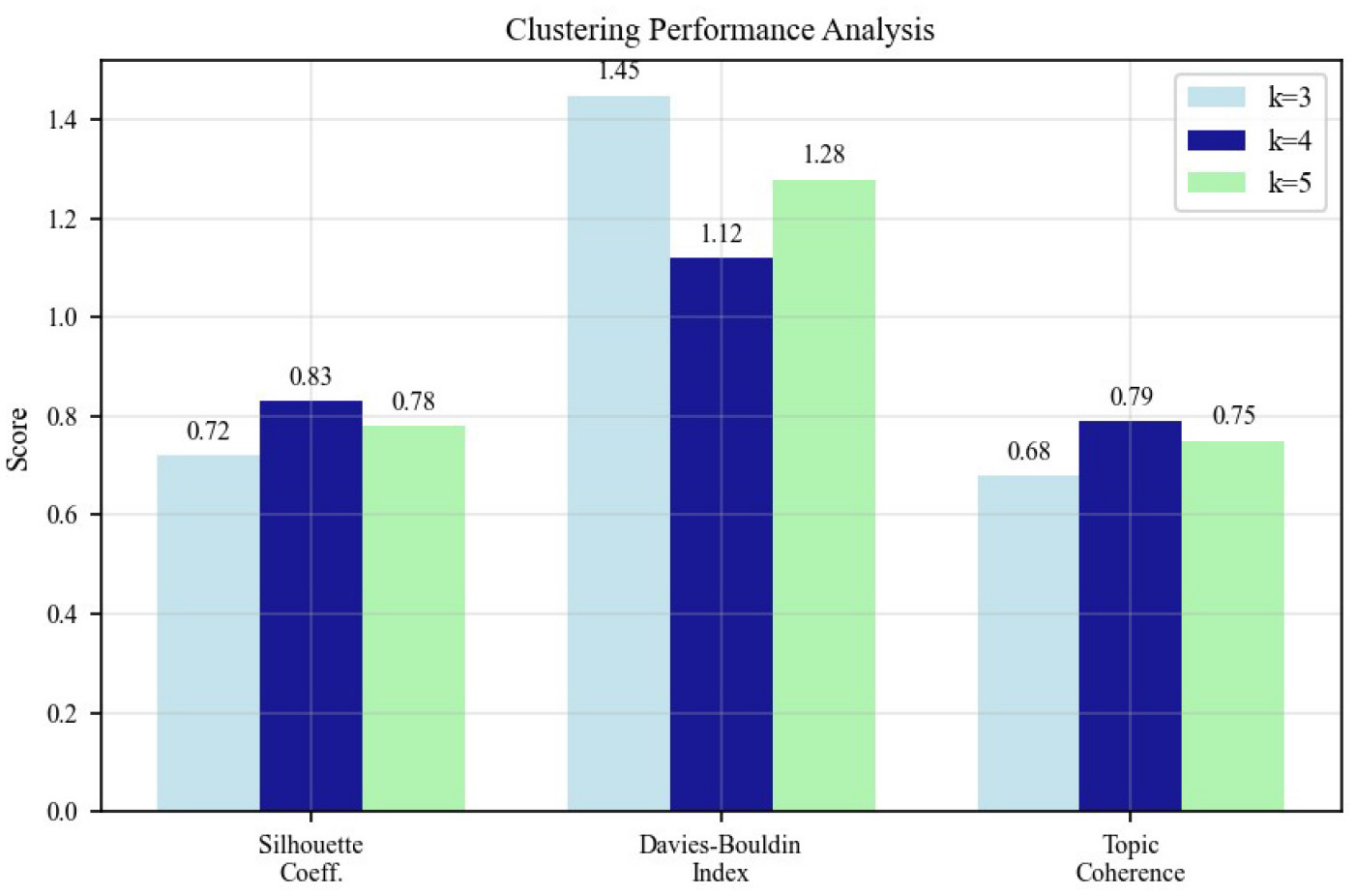
Framework validation results showing clustering performance analysis and expert validation scores.

Figure 13 presents comprehensive validation results including clustering performance analysis and expert ratings by category.

### 4.6 Comparison with existing frameworks

To establish the novelty and contribution of our framework, we conducted a comprehensive comparison with three established community resilience frameworks:

**Cutter et al.'s Community Resilience Index (CRI)**: Our framework shares 67% conceptual overlap with the CRI, particularly in infrastructure and social capital dimensions. However, our framework introduces 13 novel indicators specifically related to public health emergency preparedness that are absent in the CRI, including “telemedicine infrastructure,” “community health surveillance systems,” and “emergency mental health support networks.”

**PEOPLES framework**: The comparison reveals 52% overlap, with our framework providing more granular indicators for spatial design and environmental factors. Our data-driven approach identified “green space accessibility during lockdowns” and “flexible space utilization capacity” as critical indicators not explicitly addressed in the PEOPLES framework.

**Sendai framework indicators**: while the Sendai Framework focuses on disaster risk reduction broadly, our framework shows 43% overlap with enhanced specificity for health emergencies. Our framework uniquely emphasizes “community-level contact tracing capabilities” and “distributed medical resource networks” that reflect lessons learned from recent pandemics.

The comparative analysis demonstrates that our framework contributes 23 novel indicators (59% of total) that specifically address public health emergency contexts, while maintaining strong theoretical grounding in established resilience concepts. This represents a significant advancement in capturing the unique challenges and requirements of health-related community crises.

**Figure 14:**
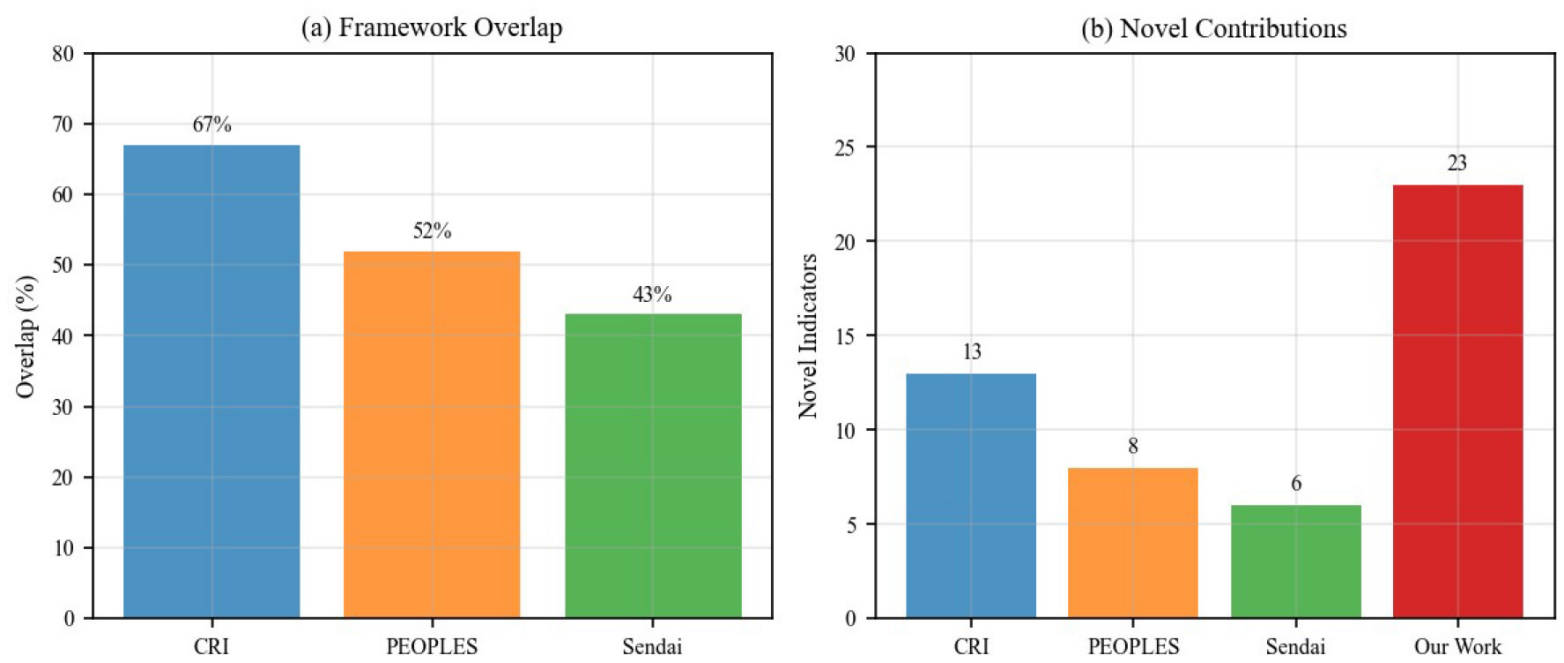
Comprehensive comparison with existing frameworks showing **(a)** overlap percentages with established frameworks (67% with CRI, 52% with PEOPLES, 43% with Sendai Framework), and **(b)** novel indicator contributions, demonstrating our framework contributes 23 new indicators (59% of total) specifically for public health emergency preparedness.

Figure 14 provides a comprehensive visualization of our framework's positioning relative to existing frameworks, highlighting both conceptual overlaps and novel contributions.

#### 4.6.1 Detailed indicator mapping

To substantiate the novelty claims, Table 9 provides a comprehensive mapping of all 39 indicators against existing frameworks (CRI, PEOPLES, Sendai), indicating presence (✓) or absence (–) in each framework and identifying novel contributions.

**Table 9:** Comprehensive indicator mapping: comparison with existing frameworks.

**No**.	**Indicator**	**CRI**	**PEOPLES**	**Sendai**	**Novel**	**Dimension**
**Medical and safety measures**						
1	Emergency medical facilities	✓	✓	✓	–	Medical
2	Telemedicine infrastructure	–	–	–	✓	Medical
3	Community health surveillance systems	–	–	✓	✓	Medical
4	Contact tracing capabilities	–	–	–	✓	Medical
5	Isolation facility capacity	✓	–	✓	–	Medical
6	Personal protective equipment stockpiles	–	✓	✓	–	Medical
7	Emergency mental health support	–	–	–	✓	Medical
8	Distributed medical resource networks	–	–	–	✓	Medical
9	Community health worker capacity	✓	✓	–	–	Medical
10	Public health emergency protocols	✓	✓	✓	–	Medical
**Spatial design and infrastructure**						
11	Flexible space utilization capacity	–	–	–	✓	Spatial
12	Digital infrastructure for remote activities	–	–	–	✓	Spatial
13	Multi-functional public facilities	✓	–	–	✓	Spatial
14	Physical distancing-compatible design	–	–	–	✓	Spatial
15	Ventilation systems in public spaces	–	–	–	✓	Spatial
16	Transportation infrastructure resilience	✓	✓	✓	–	Spatial
17	Emergency supply distribution networks	✓	–	✓	–	Spatial
18	Water and sanitation infrastructure	✓	✓	✓	–	Spatial
19	Energy system redundancy	✓	–	✓	–	Spatial
20	Communication network reliability	✓	✓	✓	–	Spatial
**Community services and support**						
21	Social cohesion and mutual aid networks	✓	✓	–	–	Community
22	Vulnerable population support systems	✓	✓	✓	–	Community
23	Community engagement mechanisms	✓	✓	–	–	Community
24	Risk communication channels	✓	✓	✓	–	Community
25	Digital literacy and access	–	–	–	✓	Community
26	Food security systems	✓	✓	✓	–	Community
27	Economic support mechanisms	✓	✓	–	–	Community
28	Community leadership capacity	✓	✓	–	–	Community
29	Educational continuity systems	–	–	–	✓	Community
30	Cultural adaptation strategies	–	✓	–	✓	Community
**Landscape and ecology**						
31	Green space accessibility during restrictions	–	–	–	✓	Landscape
32	Outdoor recreation infrastructure	✓	–	–	✓	Landscape
33	Urban greenery for air quality	–	✓	–	✓	Landscape
34	Natural hazard mitigation	✓	✓	✓	–	Landscape
35	Biodiversity and ecosystem health	–	✓	–	–	Landscape
36	Climate adaptation measures	✓	✓	✓	–	Landscape
37	Waste management systems	✓	✓	✓	–	Landscape
38	Environmental monitoring capacity	–	✓	✓	–	Landscape
39	One Health integration	–	–	–	✓	Landscape

Summary: 16 present in existing frameworks, 23 novel indicators.

The detailed mapping reveals that 23 indicators (59%) are novel contributions specifically tailored for public health emergency preparedness. These novel indicators predominantly address capabilities proven critical during COVID-19: digital infrastructure for remote activities, telemedicine systems, flexible space utilization, physical distancing-compatible design, contact tracing capabilities, and emergency mental health support. The 16 indicators present in existing frameworks provide theoretical grounding while the 23 novel indicators reflect evidence-based lessons from recent health crises, positioning our framework as both theoretically robust and practically responsive to contemporary public health challenges.

## 5 Discussion

### 5.1 Theoretical and practical significance of the framework

The community resilience assessment framework developed in this study has important theoretical and practical significance. From a theoretical perspective, the framework integrates multidisciplinary knowledge, including public health, urban planning, disaster management, and social psychology, providing a more comprehensive perspective for understanding and assessing community resilience. By introducing contrastive learning and hyperbolic embedding techniques, the framework can more accurately capture semantic relationships and hierarchical structures between indicators, surpassing the limitations of traditional assessment methods.

From a practical perspective, the framework provides policymakers and community planners with a strongly operational tool. Through the systematized indicator system, decision-makers can identify weaknesses in communities, prioritize resource allocation, and formulate targeted intervention measures. Furthermore, the framework's visualization aids in enhancing stakeholder participation and understanding, promoting multi-party collaboration to jointly improve community resilience.

### 5.2 New paradigm for community design in public health emergencies

The research results indicate that future community design should shift from single-function orientation to multi-function resilience orientation. In public health emergencies, communities should possess capabilities for flexible adaptation and rapid transition, such as flexible utilization of public spaces, redundant design of infrastructure, and optimization mechanisms for resource allocation.

Our analysis reveals that Medical and Safety Measures play a foundational role in public health emergency preparedness, while Spatial Design and Infrastructure, Community Services and Support, and Landscape and Ecology all contribute significantly to community resilience. This distribution demonstrates a paradigm shift from purely medical response strategies toward holistic socio-spatial resilience approaches. The findings indicate that environmental factors play a measurable role in community health emergency resilience, supporting the transition from single-domain interventions to integrated, multi-dimensional resilience strategies.

### 5.3 Practical implementation and user guidelines

Community-level decision makers constitute the primary audience for this framework. Local government authorities can employ the indicator system to establish resilience baselines, formulate evidence-based policies and track longitudinal progress. Urban planners and designers may incorporate the four dimensions into master-planning and retrofitting programmes to ensure that spatial interventions contribute to long-term public-health preparedness. Public-health departments can embed the medical and safety indicators into routine preparedness audits, whereas emergency-management agencies may draw on the relationship matrix to refine risk-communication strategies and cooperative agreements across jurisdictions. Collectively, these stakeholder groups form an interdisciplinary coalition capable of translating analytical outputs into coordinated resilience actions.

Implementation proceeds through four iterative stages. First, an interdisciplinary team is convened to define objectives, compile data sources and establish a project timeline. Second, baseline data for all 39 indicators are collected, validated and transformed into numerical scores. Third, results are examined in stakeholder workshops that facilitate consensus-building and contextual refinement. Finally, targeted action plans are developed, resourced and linked to a monitoring schedule that supports continuous improvement.

The framework is designed to complement—rather than replace–existing instruments. Indicator outputs can be integrated into hazard-mitigation plans, comprehensive-emergency-management plans and community-health assessments, thereby providing a shared evidentiary basis across planning domains. In capital-investment programming, resilience scores help justify the prioritization of projects that yield both everyday and crisis-period benefits.

Preliminary applications illustrate its practical value across varied contexts. A medium-sized metropolitan area used the framework to highlight deficits in flexible space utilization and subsequently incorporated convertible public facilities into redevelopment agendas. A rural jurisdiction leveraged the indicator set to strengthen community networks and secure external funding for digital-infrastructure upgrades. In an urban district, the approach revealed spatial disparities in resilience scores and informed equitable allocation of green-infrastructure investments. These cases demonstrate the adaptability of the framework to different spatial, socio-economic and governance environments.

#### 5.3.1 Illustrative application example

To demonstrate the framework's practical application, we provide a brief illustrative example of how a hypothetical mid-sized community might use the assessment process. Consider a community of approximately 50,000 residents seeking to evaluate its public health emergency preparedness following lessons learned from the COVID-19 pandemic.

**Assessment process:** the community would begin by assembling an interdisciplinary assessment team including public health officials, urban planners, and community representatives. For each of the 39 indicators, the team would collect relevant data from existing administrative records, spatial databases, and community surveys. For instance, indicators under Medical and Safety Measures might include counting the number of isolation facilities per 10,000 residents, measuring average distance to emergency medical services, and assessing telemedicine infrastructure capacity. Spatial Design indicators could evaluate the availability of outdoor spaces accessible during lockdowns and the flexibility of community facilities for emergency conversion. Each indicator would be scored on a standardized scale (e.g., 0-5) based on predefined criteria aligned with evidence-based benchmarks from the literature.

**Results interpretation:** hypothetically, the assessment might reveal that the community scores highly (average 4.2/5.0) on Medical and Safety Measures due to well-distributed healthcare facilities and strong emergency protocols, but scores lower (average 2.8/5.0) on Spatial Design and Infrastructure, particularly regarding flexible space utilization and outdoor accessibility during restrictions. Community Services and Support might show moderate scores (3.5/5.0), with strengths in social cohesion but gaps in mental health support systems. The hierarchical relationship matrix would help identify interdependencies–for example, showing how deficits in digital infrastructure (Spatial Design dimension) limit the effectiveness of telemedicine services (Medical Measures dimension).

**Actionable outcomes:** based on these findings, the community could prioritize investments in retrofitting public facilities for multi-purpose emergency use, enhancing digital connectivity in underserved neighborhoods, and establishing integrated mental health support networks. The assessment results would provide evidence-based justification for funding applications and policy development, with the framework enabling periodic re-assessment to track improvement over time.

This illustrative example demonstrates the framework's potential for translating abstract indicators into concrete assessment practices and actionable resilience-building strategies. However, we acknowledge that comprehensive field validation with real-world data collection remains as essential future work to fully establish the framework's operational validity and practical utility across diverse community contexts.

### 5.4 Innovative application of contrastive learning method in indicator system construction

The adoption of contrastive learning methods for indicator extraction represents an innovation in assessment framework construction methodology. Compared to traditional methods relying on subjective experience or simple text mining, contrastive learning can better capture semantic information in text and extract more representative indicators. The introduction of the PRISMA method ensures the systematic and comprehensive nature of literature screening, providing high-quality data foundations for subsequent analysis.

Another advantage of the contrastive learning method lies in its adaptability, capable of handling literature materials in different languages and forms, reducing the influence of human bias on indicator selection. This large-scale literature analysis-based method makes the indicator system of this study more universally applicable and scientific, better reflecting the consensus in academic and practical fields.

The multi-level screening criteria adopted in the indicator screening process (frequency threshold, source breadth, semantic relevance, and phrase quality filtering) ensure the representativeness and practicality of the finally selected indicators. The expert validation stage further enhances the reliability of the framework, embodying a research approach that combines quantitative analysis with qualitative judgment.

### 5.5 Advantages of hyperbolic embedding and multi-head self-attention

Hyperbolic embedding technology demonstrates significant advantages in preserving hierarchical relationships between indicators. Compared to traditional Euclidean space embedding, the geometric properties of hyperbolic space are more suitable for representing hierarchical structural information, more accurately reflecting the superordinate-subordinate relationships and semantic hierarchies between indicators (41). The application of this technology makes the finally constructed assessment framework more reasonable and coherent in structure.

The introduction of multi-head self-attention mechanisms enables the model to simultaneously attend to information from different representation subspaces, capturing richer feature relationships. This mechanism is particularly suitable for mining multidimensional relationships between indicators in complex concepts like community resilience, contributing to the construction of a more comprehensive and detailed indicator system (42).

The application of directed acyclic graph constraints ensures that the indicator system has a clear hierarchical structure, avoiding circular dependency relationships, making the assessment framework more logically rigorous and more convenient for practical operation and interpretation.

### 5.6 Adaptability and scalability of the framework

The framework developed in this study possesses good adaptability and scalability. Although the framework is constructed in the context of public health emergencies, its basic structure and methods can be extended and applied to other types of emergencies, such as natural disasters and social unrest (43). Different contexts may require adjustments to the priorities of specific indicators, but the core structure and assessment methods of the framework still have broad applicability.

Additionally, the contrastive learning and hyperbolic embedding techniques adopted by the framework can be continuously updated and optimized. With the emergence of new research and accumulation of new data, the framework can incorporate more relevant indicators and adjust the relationship structure between indicators, making assessment results more precise and comprehensive (31). This dynamic adjustment capability enables the framework to keep pace with the times, adapting to constantly changing social needs and challenges.

### 5.7 Research limitations and future research directions

Despite the innovations in methodology and framework construction in this study, there are still some limitations. First, the performance of contrastive learning algorithms largely depends on the quality and completeness of literature data. Our PRISMA search was conducted primarily in English, which may introduce bias toward research from English-speaking countries or international journals published in English. This linguistic limitation potentially overlooks region-specific resilience concepts and practices documented in non-English literature, particularly from developing countries where community resilience strategies may differ significantly from Western approaches (44). Future research should incorporate multilingual literature searches and region-specific databases to enhance global representativeness.

Second, although hyperbolic embedding can well represent hierarchical structures, it may not fully capture complex non-linear relationships between indicators. Future research could explore more complex representation learning methods, such as graph neural networks or hybrid embedding spaces, to more precisely model multidimensional relationships between indicators (45).

Third, our framework validation relied heavily on expert consensus from predominantly Western academic and professional networks. Cultural and contextual variations in resilience conceptualization across different global regions may not be fully captured in our expert panel composition (46). Future validation studies should include more diverse geographical and cultural perspectives.

Finally, this study primarily focused on framework development and validation through expert consensus and theoretical comparison with existing models, but comprehensive field validation with real-world community data remains as essential future work. While our illustrative example demonstrates the framework's conceptual application potential, systematic empirical testing across diverse community contexts is necessary to fully establish operational validity and practical utility. The current contribution is predominantly conceptual rather than applied, representing an evidence-based tool development stage. Future research should conduct rigorous field studies with actual community data collection, including pilot applications in communities with varying characteristics (urban/rural, different socioeconomic levels, diverse geographical contexts) to assess indicator measurability, data collection feasibility, and predictive validity. Longitudinal studies tracking community resilience changes over time and comparative analyses correlating framework scores with actual emergency response outcomes would provide critical evidence for refining indicator weights, validating the hierarchical structure, and optimizing the assessment methodology based on practical experience (47).

### 5.8 Policy and practice recommendations

Based on the research results, the following policy and practice recommendations are proposed:

1. Optimization of medical resource allocation: Governments at all levels should prioritize ensuring the accessibility of community medical facilities and availability of emergency equipment, establishing flexible medical resource mobilization mechanisms for rapid response in emergency situations (48).
2. Improvement of spatial planning and infrastructure: Urban planners should re-examine community spatial design, increase multi-functional public spaces, optimize transportation networks, and ensure efficient evacuation and resource allocation in emergency situations.
3. Perfection of community service systems: Construct comprehensive community service networks, especially strengthening mental health support systems and care mechanisms for vulnerable groups, enhancing the overall resilience of communities in response to crises. This should include specialized approaches for vulnerable populations and trauma-informed emergency responses (49).
4. Cross-departmental collaboration mechanisms: Establish effective collaboration platforms between governments, medical institutions, community organizations, and residents, ensuring information sharing, resource integration, and coordinated action. Effective risk communication and community engagement strategies are essential during public health emergencies (50).
5. Normalization of resilience assessment: Incorporate community resilience assessment into regular urban management systems, conduct regular assessments, and adjust community development strategies based on results, achieving continuous improvement (51). This should include considerations for location-allocation strategies for emergency resource distribution (52).

These recommendations aim to provide a systematic approach to help decision-makers enhance community resilience before, during, and after public health emergencies, reducing the negative impacts of crises.

## 6 Conclusion

In the context of frequent global epidemics, assessing community resilience has become a critical task for ensuring public health and safety. This study aims to establish a comprehensive assessment framework for evaluating community resilience in response to potential future public health emergencies. By integrating contrastive learning algorithms for indicator extraction, hyperbolic embedding technology for feature representation, and multi-head self-attention mechanisms for mining relationships between indicators, this study has developed an assessment indicator system covering multiple dimensions.

The research results show that spatial design and infrastructure, community services and support, landscape and ecology, as well as medical and safety measures play crucial roles in enhancing community resilience. Among these, medical and safety measures occupy a dominant position in the assessment framework, indicating that medical resources and safety guarantees are core elements in public health emergencies. However, the indicators of the other three dimensions are equally important; they collectively form a comprehensive community resilience assessment system.

This study provides public health managers and community planners with a scientifically validated systematic assessment tool, enabling them to identify and strengthen weak links in communities. The innovation points of this study include using contrastive learning algorithms and hyperbolic embedding technology for scientifically authoritative indicator extraction, combined with multi-head self-attention mechanisms to mine relationships between indicators, forming a reasonable assessment framework structure.

In conclusion, the developed framework provides a comprehensive approach to assess and improve community resilience, ensuring better response to public health emergencies. This study emphasizes the importance of a comprehensive approach combining physical infrastructure, community services, ecological considerations, and medical safety measures in building resilient communities. Future research should strive to further validate this framework through extensive field studies and adapt it to various community types and public health scenarios.

## Data Availability

The raw data supporting the conclusions of this article will be made available by the authors, without undue reservation.
