# Correction: A community public health emergency resilience assessment framework based on contrastive learning and hyperbolic embedding

**DOI:** 10.3389/fpubh.2026.1805132

**Published:** 2026-02-27

**Authors:** Quan Wen, Mazran Ismail, Muhammad Hafeez Abdul Nasir

**Affiliations:** School of Housing, Building and Planning, Universiti Sains Malaysia (USM), Penang, Malaysia

**Keywords:** community resilience, public health emergency, assessment framework, emergency preparedness, spatial design, community services, evidence-based indicators, health crisis preparedness

In the published article, author Mazran Ismail was erroneously omitted as corresponding author. The correct corresponding authors are Quan Wen (wenquan@student.usm.my) and Mazran Ismail (mazran@usm.my).

The original version of this article has been updated.

